# Developmental Profile and Diagnoses in Children Presenting with Motor Stereotypies

**DOI:** 10.3389/fped.2016.00126

**Published:** 2016-11-23

**Authors:** Francesco Cardona, Francesca Valente, Daniela Miraglia, Caterina D’Ardia, Valentina Baglioni, Flavia Chiarotti

**Affiliations:** ^1^Dipartimento di Pediatria e Neuropsichiatria Infantile, Università degli Studi di Roma “La Sapienza”, Rome, Italy; ^2^Università degli Studi Niccolò Cusano, Rome, Italy; ^3^Dipartimento di Biologia Cellulare e Neuroscienze, Istituto Superiore di Sanità, Rome, Italy

**Keywords:** stereotypies, autistic spectrum disorder, children, repetitive behaviors, neurodevelopmental disorders, complex motor stereotypies, DSM 5

## Abstract

**Introduction:**

Motor stereotypies represent a typical example of the difficulty in distinguishing non-clinical behaviors (physiological and transient) from symptoms or among different disorders [“primary stereotypies,” associated with autistic spectrum disorder (ASD), intellectual disabilities, genetic syndromes, and sensory impairment]. The aim of this study was to obtain an accurate assessment on the relationship between stereotypies and neurodevelopmental disorders.

**Methods:**

We studied 23 children (3 girls), aged 36–95 months, who requested a consultation due to the persistence or increased severity of motor stereotypies. None of the patients had a previous diagnosis of ASD. The assessment included the Motor Severity Stereotypy Scale (MSSS), the Repetitive Behavior Scale-Revised (RBS-R), the Raven’s Colored Progressive Matrices, the Child Behavior CheckList for ages 1½–5 or 4–18 (CBCL), the Social Responsiveness Scale (SRS), and the Autism Diagnostic Observation Schedule-second edition (ADOS 2).

**Results:**

All patients were showing motor stereotypies for periods of time varying from 6 to 77 months. The MSSS showed that each child had a limited number of stereotypies; their frequency and intensity were mild. The interference of stereotypies was variable; the impairment in daily life was mild. The RBS-R scores were positive for the subscale of “stereotypic behaviors” in all children. Moreover, several children presented other repetitive behaviors, mainly “ritualistic behavior” and “sameness behavior.” All patients showed a normal cognitive level. The CBCL evidenced behavioral problems in 22% of the children: internalizing problems, attention, and withdrawn were the main complaints. On the SRS, all but one of the tested patients obtained clinical scores in the clinical range for at least one area. On the ADOS 2, 4 patients obtained scores indicating a moderate level of ASD symptoms, 4 had a mild level, and 15 showed no or minimal signs of ASD.

**Discussion:**

Motor stereotypies in children with normal cognitive level represent a challenging diagnostic issue for which a finely tailored assessment is mandatory in order to define a precise developmental profile. Thus, careful and cautious use of standardized tests is warranted to avoid misdiagnosis. Furthermore, it is hard to consider motor stereotypies, even the primary ones, exclusively as a movement disorder.

## Introduction

Stereotypies are a pattern of repetitive non-functional motor behavior that can interfere with the quality of social interactions, academic or other activities, or may result in injury (1, 2). Hand/arm flapping and waving, hand rotating, and finger wiggling are the stereotypies more frequently reported even if a wide range of other repetitive movements, sometimes accompanied by sounds or vocalizations, can be observed. Stereotypic movements generally last for seconds to minutes, tend to occur in clusters, and may appear many times throughout the day (3, 4). They are often triggered by periods of excitement, being engrossed in activities, stress, fatigue or boredom, and by daydreaming (5–7).

Stereotypies typically begin during the early developmental period, and they often represent a physiological and transient stage of development. Sixty percent of neurologically typical children show some stereotypic movements or behaviors between 2 and 5 years (8, 9). Besides the physiological ones, stereotypies are a main symptom of several developmental disorders, such as autistic spectrum disorder (ASD), intellectual disabilities (ID), genetic syndromes (Rett syndrome, Lesch–Nyhan syndrome, X-fragile syndrome, and others), and are also reported in sensory impaired individuals (10–12). In particular, stereotypies, together with other restricted, repetitive patterns of behavior, interest, or activities, represent a core symptom of ASD (13). Recent research suggests that restricted and repetitive behaviors can be subdivided into at least two subcategories, repetitive sensory motor and insistence on sameness behaviors, exhibiting different relationships with age and IQ (14). Moreover, stereotypic behaviors seem to be one of the four principal pathogenetic components in ASD, possibly differing in developmental trajectories and response to treatment (15).

If the above-mentioned conditions are ruled out, the stereotypies can be categorized as “primary” complex motor stereotypies (CMS) (16–18). Therefore, in the field of child neuropsychiatry, stereotypies represent a typical example of the difficulty in distinguishing non-clinical behaviors from symptoms or among different disorders.

In the DSM 5, the categorization into “primary” or “secondary” is not present: stereotypies are classified as stereotypic movement disorder (SMD) if they occur as a primary diagnosis or secondary to another disorder. It should be mentioned that the DSM 5 permits coding of SMD also in the presence of a neurodevelopmental disorder, adding the specifier of the associated condition (e.g., SMD associated with Lesch–Nyhan syndrome). On the other hand, when ASD is present, SMD is diagnosed only when there is self-injury or when the stereotypic behaviors are sufficiently severe to become a focus of treatment.

Some of the clinical conditions associated with stereotypies are relatively easy to diagnose. For instance, most genetic syndromes have evident phenotypic traits; moreover, in some of them, repetitive behaviors show specific profiles (19). In the same way, severe intellectual disability, sensory impairment, and low-functioning autism are conditions that can be ruled out without difficulty. On the other hand, major problems can be encountered in disentangling primary CMS from stereotypies occurring in high-functioning ASD. It should be mentioned that ASD was an exclusionary criterion in many studies on primary CMS: the children with a previous history of ASD diagnosis (based on a review of medical records) and those with overt risk of ASD (based on the Autism Spectrum Screening Questionnaire, carried out during a telephone screening) were excluded from the studies (20, 21).

From a differential diagnosis perspective, little help comes from previous studies aimed at comparing primary and secondary stereotypies. Previous studies have shown that individuals with autism had higher levels and intensity of stereotypy than individuals with mental retardation (22). These findings are consistent with other studies that have reported higher levels of stereotypy in individuals with disabilities than in age-matched individuals without disabilities (23). In a comparison study between typically developing children and patients with autism and PDD-NOS, the first ones showed constant and low levels of stereotypic behavior whereas levels increased with age in the children with autism and PDD-NOS (24). In a large cohort of 277 children, which included children with autism and non-autistic developmental disorders, Goldman and Greene (25) showed that the presence of stereotypies at preschool was more strongly linked with autism than with cognitive incompetence. Moreover, the number of stereotypies per child and the variety of stereotypies has been reported to be greater in low-functioning autism group, with head/trunk, hand/finger, and gait (e.g., spinning and pacing) stereotypies being the most frequent in this group (26). Finally, in a recent study comparing primary and secondary stereotypies, Ghosh et al. (27) found that the primary ones were simple, prevalently motor, of shorter duration, and of less frequency, whereas secondary had more vocalization, complexity, longer durations, and higher frequency; unexpectedly, worsening of stereotypies was noted in a higher percentage of the primary cases. Overall, these studies have limited usefulness when dealing with individual cases.

The aim of this study was to obtain an accurate analysis on the relationship between stereotypies and neurodevelopmental disorders. Therefore, we assessed a group of children requesting a consultation due to the presence, persistence, or increased severity of motor stereotypies.

## Materials and Methods

### Participants

We studied 23 children, aged from 36 to 95 months, consecutively enrolled between September 2015 and June 2016 at the Outpatients Division of the Department of Pediatrics and Child Neuropsychiatry of “Sapienza” University of Rome. They were referred to our childhood movement disorder unit by their pediatrician. In all cases, the consultation was requested for the presence, persistence, or increased severity of motor stereotypies.

The only inclusion criterion was the presence of motor stereotypies, observed during the consultation, reported by parents or by home videos.

Exclusion criteria for the study were:

(1) known or suspected genetic syndromes;
(2) presence of other clinical neurological signs;
(3) presence of sensory impairment;
(4) previous diagnosis of ASD.

### Procedures

After the first medical examination, eligible subjects were asked to participate in the study. Participants received a complete assessment with standardized tests, including an evaluation of cognitive profile [using Raven’s Colored Progressive Matrices (CPM)]. Parents were asked to fill in questionnaires to check for other neuropsychiatric conditions: the Child Behavior CheckList for ages 1½–5 or 4–18 (CBCL), and the Social Responsiveness Scale (SRS) for children aged over 48 months. The Motor Severity Stereotypy Scale (MSSS) and the Repetitive Behavior Scale-Revised (RBS-R) were used to assess the stereotypies. The Autism Diagnostic Observation Schedule-Second edition (ADOS 2) was administered to the patients by fully qualified personnel, blinded to the clinical diagnosis. The study was approved by the “Sapienza – University of Rome” Ethics Committee (Ref. 3477). The parents gave their informed consent at the time of enrollment in the study.

### Statistical Analysis

Quantitative data were summarized by means ± SD and categorical data by absolute and percent frequencies. Differences in total and subtotal scores of CBCL and SRS among groups based on the ADOS 2 calibrated severity score were assessed by the analysis of variance, followed by the Tukey test for multiple comparisons. The Pearson linear correlation coefficient *r* was computed to estimate the correlation between the CBCL pervasive developmental problem score and the ADOS 2 and SRS scores. To take into account possible violations of assumptions of parametric tests, non-parametric Kruskal–Wallis analysis of variance and Spearman’s rank correlation coefficient were also applied. Since the results perfectly overlapped those of parametric analyses, only the latter are presented. Statistical analyses were performed by STATA Release 8.1.

## Results

The sample consisted of 23 children – 20 boys and 3 girls – aged between 36 and 95 months (mean = 53; SD = 15). The review of medical records showed that four patients had a history of motor delay (walking alone after 18 months of age), eight had motor coordination problems or immaturity in graphomotor skills, and seven had delay in expressive language. None of the children had a previous history of ASD diagnosis. At the time of consultation, all patients were showing motor stereotypies for periods of time varying from 6 to 77 months (mean = 33; SD = 16). The onset of stereotypies was reported between 4 and 51 months of age (mean = 19; SD = 14).

Their semiology had remained unchanged over time, mostly characterized by CMS: patients presented a single repetitive movement or complex sequences involving the entire body such as jumping, kicking, flapping hands, moving hands in front of the face or the eyes, or involving movements and “dystonic” postures of the trunk. Sounds or vocalizations accompanied the motor stereotypies in three patients. From the time of their onset, increasing frequency of stereotypies has been reported. Excitement or boredom was described as common triggers.

The MSSS showed that each child had a limited number of stereotypies, between 1 and 3 (mean = 1.6); their frequency and intensity were mild (range 1–4; mean = 2.8 for both). The interference of stereotypies was variable, from 0 to 4 (mean = 1.6). The mean MSSS final score was 21.1, suggestive of a mild impairment in daily life.

On the RBS-R, items in the subscale of “stereotypic behaviors” were scored by all children; moreover, the questionnaire revealed the presence of other repetitive behaviors in several children, mainly “ritualistic behavior” and “sameness behavior,” even if at a lower degree (Table 1).

**Table 1:** **RBS-R: subscale scores and number endorsed**.

	Subscale scores (mean ± SD)	Number endorsed^a^ (mean ± SD)	Number of patients with number endorsed ≠ 0
I – stereotypic behavior	3.6 ± 2.3	2.0 ± 1.1	23
II – self-injurious behavior	0.4 ± 1.1	0.3 ± 0.6	4
III – compulsive behavior	1.1 ± 1.9	0.7 ± 1.2	9
IV – ritualistic behavior	1.4 ± 2	0.9 ± 1.1	12
V – sameness behavior	1.9 ± 2.5	1.6 ± 1.7	15
VI – restricted behavior	0.9 ± 1.5	0.5 ± 0.7	8

*^a^Number of items endorsed for each subscale (any rating other than 0)*.

All patients showed a normal cognitive level: in particular, on the Raven’s CPM, they obtained scores ranging between 32 and 95 percentiles (mean = 80, SD = 16), corresponding to IQ levels superior to 85.

The CBCL evidenced behavioral problems in 22% of the children (Table 2); internalizing problems, attention, and withdrawn were the main complaints reported by parents. Among the DSM-oriented scales, the pervasive developmental problems were the principal affected domain: indeed, three children obtained borderline and four clinical scores.

**Table 2:** **Number of patients obtaining borderline or clinical scores at the Child Behavior CheckList – ages 1½–5 or 4–18, according to chronological age**.

	Borderline	Clinical
CBCL total	2	5
CBCL internalizing problems	3	5
CBCL externalizing problems	4	2
**Symptoms scales**		
Emotionally reactive	2	1
Anxious/depressed	0	1
Somatic complaints	0	2
Withdrawn	3	2
Sleep problems	0	2
Attention problems	3	3
Aggressive behavior	1	0
**DSM-oriented scales**		
Anxiety problems	1	0
Pervasive developmental problems	3	4
ADHD problems	2	1
Oppositional defiant problems	0	0

Symptoms of ASD were assessed by the SRS questionnaire (in the 15 children older than 48 months) and by the ADOS 2.

On the SRS, all but one of the 15 patients obtained clinical scores in the clinical range at least in one area (Table 3). Obviously, the more frequently affected domain was “restricted interests and repetitive behavior,” which indicated the clinical range in 11 out of 15 children. Moreover, “social motivation” and “SRS total score” were affected in 56% of children.

**Table 3:** **Number of patients obtaining clinical scores at the Social Responsiveness Scale (SRS)**.

	Mild range (*T* score: 60–65)	Moderate range (*T* score: 66–75)	Severe range (*T* score >76)
Total score	4	1	3
Social awareness	1	4	2
Social cognition	2	2	1
Social communication	3	2	1
Social motivation	5	3	1
Restricted interests and repetitive behavior	2	4	5

For the ADOS 2, four patients obtained scores indicating a moderate level of ASD symptoms (calibrated severity score = 6–7), four had a mild level (calibrated severity score = 4–5), and 15 showed no or minimal signs of ASD (calibrated severity score = 0–3).

In the whole sample, no correlation was found between the ADOS 2 scores (calibrated severity score, total, and subtotal raw score) and the measures of stereotypic behavior (MSSS and RBS-R scores, subtotal, and total).

No statistical differences were found between subgroups of patients – divided on the basis of the ADOS 2 calibrated severity score – with regard to the total and subtotal scores of CBCL and SRS (Table 4). However, in the 15 patients aged above 48 months, a moderate correlation was found between the CBCL pervasive developmental problem scores and the following SRS scores: total (*r* = 0.370) and social communication (*r* = 0.585).

**Table 4:** **Comparison of CBCL and SRS *T* scores and number of patients with clinical scores in subgroups (following ADOS 2 score)**.

ADOS 2 – calibrated severity score	0–3	4–5	6–7
Number of patients	15	4	4
Mean age (range) (months)	57 (36–95)	37 (36–39)	57 (55–60)
**CBCL**			
Total score	Mean = 57	Mean = 53	Mean = 57
	B = 1; C = 4	B = 1; C = 0	B = 0; C = 1
PDD subscale	Mean = 57	Mean = 60	Mean = 65
	B = 0; C = 3	B = 0; C = 1	B = 3; C = 0
**SRS**			
Total score	Mean = 61		Mean = 61
	M = 3; Mo = 1; S = 2		M = 1; Mo = 0; S = 1
Social awareness	Mean = 63		Mean = 59
	M = 1; Mo = 2; S = 2		M = 0; Mo = 2; S = 0
Social cognition	Mean = 57		Mean = 56
	M = 1; Mo = 1; S = 1		M = 1; Mo = 1; S = 0
Social communication	Mean = 59		Mean = 54
	M = 2; Mo = 1; S = 1		M = 1; Mo = 1; S = 0
Social motivation	Mean = 58		Mean = 65
	M = 2; Mo = 2; S = 1		M = 3; Mo = 1; S = 0
Restricted interests/repetitive behaviors	Mean = 71		Mean = 70
	M = 1; Mo = 3; S = 4		M = 1; Mo = 1; S = 1

*B, borderline; C, clinical; M, mild; Mo, moderate; S, severe*.

## Discussion

In this study, we focused on the assessment of a group of children seeking medical attention for the presence or the persistence of motor stereotypies. According to our data, the impairment due to the stereotypies was mild, without a clear interference in daily activities. The referral of children with mild stereotypies could be due to parents’ concerns about a movement disorder that lasts over time and/or to an increased sensitivity to the ASD issue. Some of these children have a history of slight developmental delay in some areas (motor or language), but all had a normal cognitive level and no previous diagnosis of ASD.

Based on our assessment, most of them – 65% of the sample – can be easily diagnosed as having primary CMS. Namely, they showed a few stereotypies, with mild impairment; moreover, the ADOS 2 evidenced no or minimal risk of ASD. Notably, in many of these children, the RBS-R questionnaire disclosed the presence of other symptoms, mainly ritualistic behaviors, sameness, and restricted interests.

These features constitute core symptoms of ASD and their presence, even if at a low level, in our “primary” patients, highlights the difficulty to define the boundaries between transient stereotypic movements in otherwise typically developing children, the primary motor stereotypies with or without comorbidity, and the stereotypic movements in the course of ASD.

Moreover, in some previous studies on children with primary CMS (4, 20), a series of comorbid conditions have already been reported; in particular, ADHD, tic disorders, developmental coordination disorder (DCD), and other neuropsychological problems were described in a significant percentage of cases. Thus, as the DSM 5 clearly states, the presence of stereotypic movements may indicate an undetected neurodevelopmental problem. Among these comorbidities, DCD seems to take on great interest, being present in school age (20) as well as preschool age patients (Baglioni et al., in preparation).

On the basis of all these observations, it is difficult to consider motor stereotypies, even the primary ones, exclusively as a movement disorder (28). A limited number of our patients showed behaviors compatible with a diagnosis of non-autistic ASD or autism, following the calibrated severity score of ADOS 2. This index is considered to be reliable and stable over time (29). These findings were unexpected mainly because, in the past, many of our patients underwent clinical consultations for developmental delay and the diagnosis of ASD was never suspected. A possible explanation for this could be that problems of communication or socialization were overlooked due to their normal cognitive level and/or that subsequent evaluations might be sensitive to developmental changes in social and communication goals that have to be progressively attained during development.

On the other hand, it should be noted that in the whole sample, important discrepancies between the results of different tests were disclosed. In particular, high rates of clinical scores on the SRS were also found in patients who showed no or minimal risk of ASD according to the ADOS 2.

The SRS is an instrument developed to measure social impairment, but many items also describe other core features of ASD, including communication deficits and repetitive behaviors (30), as well as symptoms not exclusively related to ASD diagnostic criteria (31). Moreover, when using the SRS as a quantitative phenotype measure, the effects of non-ASD-specific factors must be considered; if not, SRS scores are more appropriately interpreted as indicating general levels of impairment, rather than severity of ASD-specific symptoms or social impairment (32).

Our study has some limitations. First of all, a small number of children participated. Second, the wide age range of the subjects hampered gathering more homogeneous data (i.e., the SRS is validated from the age of 4 years, while the SRS-2, which can be administered from the age of 1.5 years, is not yet available in Italian). Third, we did not use the Autism Diagnostic Interview that, together with the ADOS 2 and the criteria of DSM 5, could have allowed us to gain more firm diagnostic conclusions within the study.

Nonetheless, our study highlights the challenge of establishing a categorical diagnosis in children with motor stereotypies. Obviously, beyond the classification, this issue is important in terms of treatment of stereotypies that is still a debated problem (33–36).

Waiting for the results of studies investigating the pathophysiological aspects of stereotypies in ASD subjects as in “primary” cases and possibly supporting current hypotheses (37–41), a dimensional approach to the diagnosis of stereotypic behaviors could be particularly suitable. In this perspective, the view of the DSM 5, that puts together the stereotypies (with the exceptions described above) and permits coding additional conditions, seems to be more useful than the rigid categorization of “primary” and “secondary.”

In conclusion, complex stereotypies in children with normal cognitive level represent a challenging diagnostic issue for which a finely tailored assessment is mandatory in order to evaluate their peculiar developmental sentinel role. Notably, a careful and cautious use of standardized tests is warranted to avoid misdiagnosis.

## Author Contributions

FC made a substantial contribution to the conception of the work and in writing the paper; FV, DM, and VB contributed to the acquisition of data; CD contributed to the interpretation of data and literature analysis; FCh contributed to statistical analysis and critically reviewed the manuscript. All the authors read and approved the final version of the manuscript.

## Conflict of Interest Statement

The authors declare that the research was conducted in the absence of any commercial or financial relationships that could be construed as a potential conflict of interest.
